# Efficacy and safety of indigo naturalis oil extract (Lindioil ointment) for the treatment of atopic dermatitis: a randomized, crossover, evaluator-blinded, controlled trial

**DOI:** 10.3389/fphar.2025.1546589

**Published:** 2025-07-08

**Authors:** Chin-Yi Yang, Chun-Bing Chen, Chun-Wei Lu, Min-Hui Chi, Jennifer Wu, Wen-Hung Chung, Be-Han Lee, Yin-Ku Lin

**Affiliations:** ^1^ Department of Dermatology, New Taipei Municipal Tucheng Hospital, New Taipei City, Taiwan; ^2^ Department of Dermatology, Drug Hypersensitivity Clinical and Research Center, Chang Gung Memorial Hospital, Keelung, Taiwan; ^3^ College of Medicine, Chang Gung University, Taoyuan, Taiwan; ^4^ Chang Gung Immunology Consortium, Chang Gung Memorial Hospital, Taoyuan, Taiwan; ^5^ Department of Dermatology, Chang Gung Memorial Hospital, Keelung, Taiwan; ^6^ Department of Traditional Chinese Medicine, Chang Gung Memorial Hospital, Keelung, Taiwan; ^7^ School of Traditional Chinese Medicine, Chang Gung University, Taoyuan, Taiwan

**Keywords:** atopic dermatitis, body surface area (BSA), dermatology life quality index (DLQI) eczema areas severity index (EASI), indigo naturalis, Lindioil, pruritus numeric rating scale (NRS), tacrolimus

## Abstract

**Introduction:**

Lindioil ointment or its compound formulations are commonly used traditional Chinese medicine practitioners to treat adult eczema or localized dermatitis. This study aimed to determine the efficacy and safety of Lindioil ointment (indigo naturalis oil extract) for treating atopic dermatitis (AD).

**Methods:**

This was a prospective, randomized, crossover, evaluator-blinded, controlled study. Twenty-two patients with a median age of 26.5 (range, 20.8–44.3) years were treated with Lindioil or tacrolimus 0.1%. The primary outcome was change in the eczema areas severity index (EASI), body surface area (BSA), pruritus numeric rating scale (NRS) and dermatology life quality index (DLQI) after each 6-week treatment.

**Results:**

After 6 weeks of treatment, the EASI decreased significantly from 6.6 to 3.4 (P = 0.017) in the Lindioil group, and from 6.7 to 1.9 (P < 0.001) in the tacrolimus group. The BSA percentage change was significantly less in the tacrolimus group (−43.6% vs. −86.7%, P = 0.002). Significant differences between the 2 groups were observed in NRS (−2.5 vs. −5.5, P = 0.005) and DLQI median change (−5 vs. −10, P = 0.005). After Lindioil or tacrolimus ointment therapy, AD lesions' skin microbiota shifted from Firmicutes dominance to Proteobacteria dominance, resembling non-lesion skin. The proportion of *Staphylococcus aureus* species in AD lesions significantly decreased after both treatments, and was not different from that of non-lesion skin.

**Discussion:**

Lindioil ointment is effective for the treatment of mild-to-severe AD and has less side effects compared to tacrolimus. Lindioil ointment may alleviate AD by altering skin microbiota.

**Clinical Trial Registration:**

The study was registered in ClinicalTrials.gov, under the number NCT03614221.

## 1 Introduction

Atopic dermatitis (AD), also referred to as atopic eczema, is one of the most common chronic inflammatory skin diseases globally, with a lifetime prevalence ranging between 15% and 20% (Weidinger et al., 2018). In the past few decades, the prevalence of AD in Taiwan has increased significantly, ranging from 4.1% to 6.7% (Chan et al., 2021). Patients often experience severe skin itching, leading to insufficient sleep which negatively affects school, work performance, and overall quality of life.

The first-line treatment for AD is topical steroid ointments. Although these medications demonstrate good efficacy, long-term use may cause side effects such as skin atrophy, vasodilation, and adverse effects on adrenaline secretion which can affect growth. Second-line medications are immunosuppressant such as tacrolimus 0.1% ointment and pimecrolimus ointment. These medications do not have steroid-related side effects; however, their long-term use may pose an increased risk of lymphoma. This causes many patients and their families to seek alternative therapies that are effective and have fewer side effects, such as traditional Chinese medicine.

Indigo naturalis (Qingdai) is a herbal medicine extracted from the leaves or stems of various plants, including *Baphicacanthus cusia* (Nees) Bremek, *Polygonum tinctorium*, *Isatis indigotica*, and *Indigofera tinctoria* (Qi-Yue et al., 2020). Indigo naturalis has a long-standing history of application in traditional Chinese medicine, attributed to its antipyretic, anti-inflammatory, antiviral, antimicrobial, and detoxifying properties. In 2008, we optimized the formulation for indigo naturalis and named it “Lindioil ointment” that has achieved patents in the United States, the European Union, Taiwan, China, and other countries. Our previous clinical trials have demonstrated its efficacy and safety in treating psoriasis and psoriatic nails (Lin et al., 2015; Lin et al., 2011; Lin et al., 2012a; Lin et al., 2014; Lin et al., 2018). Notably, Lindioil ointment, or its compound formulations, are commonly used by traditional Chinese medicine practitioners to treat adult eczema and localized dermatitis. In our previous randomized, double-blind, placebo-controlled clinical trial, patients with AD showed an average percentage decrease of about 50% in the Eczema Area Severity Index (EASI) in the Lindioil group compared with an average percentage decrease of 20% in the placebo group (Lin et al., 2020). No treatment-related adverse events were observed during the study period (Lin et al., 2020).

An increasing number of studies have demonstrated dysbiosis in the skin microbiota of patients with AD. In lesional and non-lesional skin, and nasal passages the diversity of microbial communities is consistently lower in patients with AD compared with healthy control groups, and there is a significant negative correlation of diversity with the severity of AD (Clausen et al., 2018). In particular, large colonization of *Staphylococcus aureus* are observed during a severe relapse of AD, exacerbating skin inflammation (Bjerre et al., 2017). Treatment with topical tacrolimus 0.1% for 3 weeks has been shown to significantly reduce the colonization of *S. aureus* (Pournaras et al., 2001). Recent studies have also revealed that after 4 weeks of topical tacrolimus treatment, colonization rates of certain symbiotic bacteria such as *Dermacoccus*, *Pseudomonas*, and *Corynebacterium* are significantly increased, thereby exerting a positive impact on the skin microbiota of patients with AD (Wongpiyabovorn et al., 2019). Notably, the main components of Lindioil ointment, Qingdai and indirubin, have antimicrobial properties and inhibit the growth of Gram-positive bacteria, including *S. aureus*, as well as fungi such as *Aspergillus fumigates* and *Candida albicans* (Ponnusamy et al., 2010; Chiang et al., 2013; Gaitanis et al., 2019).

To date, the association between the efficacy of Lindioil ointment and skin microbiota in patients with AD is not fully unknown. Thus, the purpose of this study was to further determine the efficacy and safety of Lindioil ointment for the treatment of AD, and also investigate the association between the efficacy of Lindioil ointment and skin microbiota.

## 2 Materials and methods

### 2.1 Study design

This was a prospective, randomized (with crossover), evaluator-blinded, controlled trial conducted at 2 medical centers from June 2019 to December 2021. The study consisted of a screening phase of up to 1 week, a first treatment phase of 6 weeks, a washout phase of 4 days–8 weeks (Breneman et al., 2008; Rubins et al., 2005; Undre et al., 2009), a crossover second treatment phase of 6 weeks, and a follow-up phase of 4 days–8 weeks. Patients with mild-to-severe AD meeting the inclusion and exclusion criteria of the study were randomly assigned to receive either Lindioil ointment or tacrolimus ointment 0.1% during their second visit to the clinic (considered baseline and may coincide with the screening phase visit).

This study was conducted in compliance with the ethical principles of the Declaration of Helsinki and Good Clinical Practice Guidelines, and was approved by the Institutional Review Board of Chang Gung Memorial Hospital (approval number: 201800023A0). This study was also registered at Clinical Trials.gov, number NCT03614221.

### 2.2 Study population and procedures

**FIGURE 1:**
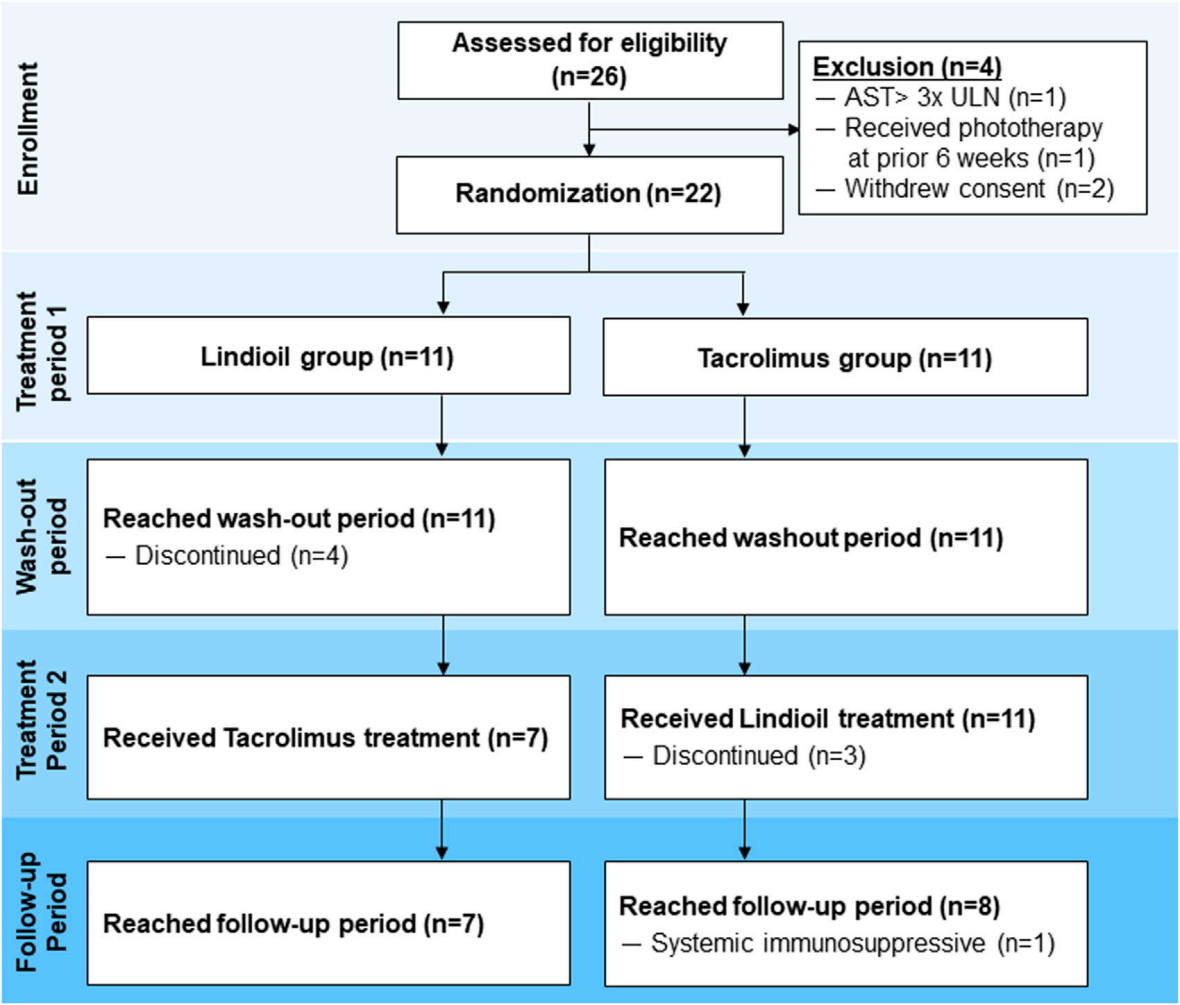
Flow diagram of patient inclusion.

Patients 6–65 years old with mild-to-severe AD who met the United Kingdom (UK) diagnostic criteria of AD, with lesions covering a total body surface area (BSA) of 3%–40%, and an Investigator’s Global Assessment (IGA) score of 2–4 were eligible for inclusion (Figure 1). Patients experiencing an acute episode of AD and those with concurrent bacterial or viral infections were excluded. Other exclusion criteria were: 1) Allergy to Lindioil ointment, tacrolimus ointment, or their excipients; 2) Received systemic treatments (e.g., immunosuppressants) 14 days before the present trial; 3) Received light therapy (UVB or PUVA) within the previous 42 days; 4) Received topical anti-dermatitis drugs within 4 days before the present trial; 5) The presence of serious medical conditions such as severe and poorly controlled chronic diseases (e.g., uncontrolled hypertension, diabetes, gout, and hyperthyroidism); 6) Remarkable abnormalities in liver or kidney function based on laboratory tests within the 30 days before the baseline visit (e.g., aspartate aminotransferase or alanine aminotransferase >3 times the upper limit of normal, or creatinine >2.0 mg/dL, or at the investigator’s discretion for clinically significant abnormalities in blood test values). Female patients who were currently lactating or pregnant, or intended to become pregnant during the trial period were also excluded.

After applying the inclusion and exclusion criteria, 22 patients (11 treated with Lindioil ointment and 11 with tacrolimus ointment) were included. All patients completed the 1st treatment period, and 7 patients completed the 2nd treatment period with Lindioil ointment and 7 completed the 2nd treatment period with tacrolimus ointment. However, among the patients who completed the 1st treatment period, 4 patients treated with Lindioil ointment discontinued the study during the wash-out period. Of the 2nd treatment period with tacrolimus, 3 patients discontinued the study during the 2nd treatment period and 1 patient was treated with a systemic immunosuppressive during the follow-up period. Finally, the efficacy of 14 patients with Lindioil ointment and 14 patients with tacrolimus ointment was compared. The safety analysis consisted of 22 patients treated with Lindioil ointment and 18 patients treated with tacrolimus ointment.

### 2.3 Drug preparation and treatment

Lindioil ointment is a trademark product name created by the author using a proprietary extraction and formulation process which meets consistent commercial pharmaceutical Chemistry, Manufactory and Control (CMC) standards. It has obtained the European patent application about “oil-extracted product of indigo naturalis, and preparation process and use thereof” (EP2489358A1, EP2489358B1).

Lindioil ointment was prepared by the Chuang Song Zong (CSZ) Pharmaceutical Co., Ltd. (Taiwan). The preparation and verification of the quality of Lindioil ointment, including HPLC-fingerprint of Lindioil ointment, were described in our previous studies (Lin et al., 2014; Lin et al., 2020). Tacrolimus ointment was purchased from Leo Pharma A/S, Ballerup, Denmark.

During each treatment period, Lindioil ointment (0.5 g per time) or tacrolimus ointment (0.1 g per time) was evenly applied to the AD lesion (10 cm × 10 cm) twice a day, once in the morning and once in the evening, with an interval of approximately 12 h between applications.

### 2.4 Efficacy and safety endpoints

The primary efficacy endpoint was the average percentage change in EASI score (ranging from 0 to 72) after each 6-week treatment period. The secondary efficacy endpoints were: 1) The proportions of patients achieving improvements of 50%, 75%, and 90% in EASI score (EASI-50, EASI-75, EASI-90) after the completion of each 6-week treatment period; 2) The proportion of patients achieving complete (IGA = 0) or near complete (IGA = 1) resolution after each 6-week treatment period; 3) The average percentage change in the area of dermatitis relative to the total BSA before and after each 6-week treatment period; 4) The number of days from treatment termination to recurrence (IGA ≥2) for patients who achieved IGA = 0 or 1 after the 6-week treatment period (Lin et al., 2011); Change in the pruritus Numeric Rating Scale (pruritus NRS, 0–10) scores for itching before and after each 6-week treatment period (Lin et al., 2012a); Change in the Dermatology Life Quality Index (DLQI) scores before and after each 6-week treatment period (Lin et al., 2014); The proportion of patients achieving a rating of “much better” or “very much better” on the Subject’s Global Assessment (SGA, 0–6) scale after the completion of each 6-week treatment period (Lin et al., 2018); Patient preferences for Lindioil ointment or tacrolimus ointment (Lin et al., 2020); Changes in the skin microbiota before and after treatment with Lindioil ointment and tacrolimus ointment during the 6-week treatment periods. The safety endpoints included vital signs and physical examination findings, hematological and biochemical blood test results, urinalysis results, and the occurrence of adverse events.

### 2.5 Collection of superficial skin samples

Superficial skin samples were taken from 2 sites to determine the microbiota of non-lesion skin (right anterior forearm) and that of AD lesions. From subjects, the replicate swabs were taken at 2 sites. A new sterile applicator was moistened in sterile TES buffer (10 mM Tris-HCl; 1 mM EDTA; 100 mM NaCL) and used to swab the skin at the specified site 40 times over a 10 × 10 cm^2^ area, pressing firmly and twirling the swab to coat all surfaces. The applicator was then placed into a microtube with 0.5 mL of TES buffer, and rotated against the side of the vial to release any biomaterial present. Immediately after sampling, samples were labeled and frozen at −80°C until shipment for processing.

### 2.6 DNA extraction, sequencing, sequencing data processing, and species annotation

DNA extraction was carried out using an EasyPrep Stool Genomic DNA kit (Tools, New Taipei City, Taiwan), according to the manufacturer’s instructions. Full-length 16s rRNA sequences were analyzed using a PacBio Sequel II system following the protocol “Procedure and Checklist-Full-Length 16S Amplification, SMRTbell^®^ Library Preparation and Sequencing” and DADA2 R software package (Callahan et al., 2016; Callahan et al., 2019). Briefly, full-length 16s rRNA was amplified using a barcoded universal primer set (27F + 1492R) from extracted DNA samples, and purified with AMPure PB beads. Equal amounts of the purified amplicons were pooled for SMRTbell^®^ Library construction. Purified and quality-checked SMRTbell^®^ libraries were sequenced on a PacBio Sequel II system (Pacific Biosciences). Subreads with more than 3 full-passes and more than 20 read quality (RQ) were used to generate circular consensus sequences (CCSs), and only HiFi reads (CCS reads >30 RQ) were processed using the DADA2 pipeline to filter out noisy sequences, correct errors in marginal sequences, remove chimeric sequences, and eliminate singletons to infer amplicon sequence variants (ASVs). ASV tables with taxonomical classifications were generated based on the NCBI database.

### 2.7 Statistical analysis

All statistical analyses were performed using SAS version 9.4 software. Continuous variables were presented in the median and range. An independent T-test (or Mann-Whitney U test) or paired T-test (or Wilcoxon signed-rank test) was used to compare continuous data. Categorical variables were presented as count and percentage, and compared with the chi-square test (or Fisher’s exact test, if necessary) or McNemar test. Statistical significance was defined as P < 0.05.

The intent-to-treat (ITT) population was defined as all randomized patients who received at least 1 treatment of the 2 study treatments, and with assessment compared to the baseline EASI. The per-protocol (PP) population was defined as all eligible patients who received treatments without major protocol deviation (at least 80% of days), with EASI assessments at Visit 2 and Visit 8, and efficacy evaluation at Visit 6 and Visit 12. The safety population consisted of all randomized patients who received at least 1 treatment.

The primary efficacy endpoint was comparison of the post-treatment change between patients treated with Lindioil ointment and tacrolimus ointment 0.1%, using a linear mixed-effects models adjusted for age and sex, and calculation of the lower limit of a 2-sided 95% confidence interval (CI). For secondary endpoint comparisons, the paired *t*-test or Wilcoxon signed-rank test was used to compare BSA, pruritus NRS, and DLQI; the McNemar test was used to compare EASI-50, EASI-75, EASI-90, IGA 0 or 1, and SGA 0 or 1. For safety endpoints, an independent T-test or Mann-Whitney U was used for intergroup comparisons; paired T-test or Wilcoxon signed-rank test was performed for intragroup comparisons; chi-square test or Fisher’s exact test was performed for intergroup comparisons; and McNemar test was for intragroup comparisons.

Sample size analysis was performed based on the following, hypothesizing that both study treatments were continued for 6 weeks): 1) A average improvement percentage of 49.9% and 54.1% in EASI compared to baseline; 2) Intragroup standard deviation of 36.5%; (3) One-sided significance level of 0.025; (4) Power = 0.8; (5) Withdrawal rate of 20% (6) Non-inferior threshold value of 19% (Paller et al., 2005); 7); 2 crossover groups. Power Analysis Sample Size (PASS) software version 15 was used to calculate the sample size. The required number of enrolled patients was estimated as 123.

## 3 Results

### 3.1 Patient characteristics

This study intended to enroll 123 patients. However, due to the outbreak of COVID-19 only 26 patients were enrolled. Among the 26 patients, 22 were randomized in 1:1 ratio to receive either Lindioil ointment or tacrolimus ointment 0.1% as the first treatment. These 22 patients were included in the safety assessment. All 11 patients in each group completed the first treatment phase, and 7 patients (64%) in each group completed the second treatment phase. The patient disposition process, including reasons for withdrawal are summarized in Figure 1. Patient demographic and baseline clinical characteristics are summarized in Table 1. The median age of the patients was 26.5 (range 20.8–44.3) years, the median BMI was 23.0 (range 15.7–35.8) kg/m^2^, and 54.5% (n = 1 2) were males. The median age of AD onset was 10 (range 0–24) years, and median AD duration was 17.8 (range 7.1–36.3) years. Among the patients, 77% (n = 17) had personal history of allergic rhinitis or asthma, and 68% (n = 15) had a family history of AD, allergic rhinitis, asthma, or eczema. In addition, 45.5% (n = 10) had been treated with a systemic immunosuppressive such as steroids, azathioprine, or cyclosporine. At baseline, the median BSA was 19.5%, median EASI was 8.8, median IGA was 3, median pruritus NRS was 7, and median DLQI was 13.

**TABLE 1:** Patient demographic and clinical characteristics.

	Total (N=22)	Lindioil first (n=11)	Tacrolimus first (n=11)
Age (years), median (range)	26.5 (20.8–44.3)	29.1 (20.8–44.3)	24.9 (21.1–35.6)
Gender, n (%)			
Male	12 (54.5)	4 (36.4)	8 (72.7)
Female	10 (45.5)	7 (63.6)	3 (27.3)
BMI (kg/m^2^), median (range)	23.0 (15.7–35.8)	23.0 (20.5–28.9)	23.2 (15.7–35.8)
Onset age (years), median (range)	10 (0–24)	8 (0–15)	11 (0–24)
AD duration (years), median (range)	17.8 (7.1–36.3)	22.6 (10.6–36.3)	14.9 (7.1–28.6)
Atopic history, n (%)			
Allergic rhinitis or asthma	17 (77.3)	9 (81.8)	8 (72.7)
Allergies	13 (59.1)	7 (63.6)	6 (54.5)
Atopic family history	15 (68.2)	8 (72.7)	7 (63.6)
Prior treatments for AD, n (%)			
Topical corticosteroids	22 (100.0)	11 (100.0)	11 (100.0)
Topical calcineurin inhibitors	5 (22.7)	4 (36.4)	1 (9.1)
Systemic immunosuppressive treatments	10 (45.5)	4 (36.4)	6 (54.5)
Traditional Chinese Medicines	13 (59.1)	6 (54.5)	7 (63.6)
Baseline clinical characteristics			
BSA (%), median (range)	19.5 (3.7–35.0)	11.5 (3.7–35.0)	22 (7.6–33.0)
BSA> 10%, n (%)	16 (72.7)	7 (63.6)	9 (81.8)
EASI score, median (range)	8.8 (3.1–15.6)	6.7 (3.1–15.6)	9.2 (4.1–14.1)
IGA score, median (range)	3 (3, 4)	3 (3, 4)	3 (3, 4)
3, n (%)	14 (63.6)	6 (54.5)	8 (72.7)
4, n (%)	8 (36.4)	5 (45.5)	3 (27.3)
Pruritus NRS, median (range)	7 (3–10)	7 (4–10)	6 (3–9)
NRS≥ 7, n (%)	12 (54.5)	7 (63.6)	5 (45.5)
DLQI, median (range)	13 (3–23)	13 (3–22)	13 (3–23)
DLQI > 10, n (%)	14 (63.6)	7 (63.6)	7 (63.6)

AD, atopic dermatitis; BMI, body mass index; BSA, body surface area involved; EASI, eczema area and severity index; IGA, Investigator’s Global Assessment; NRS, numerical rating scale; DLQI, Dermatology Life Quality Index; SGA, Subject’s Global Assessment.

### 3.2 Efficacy results

**FIGURE 2:**
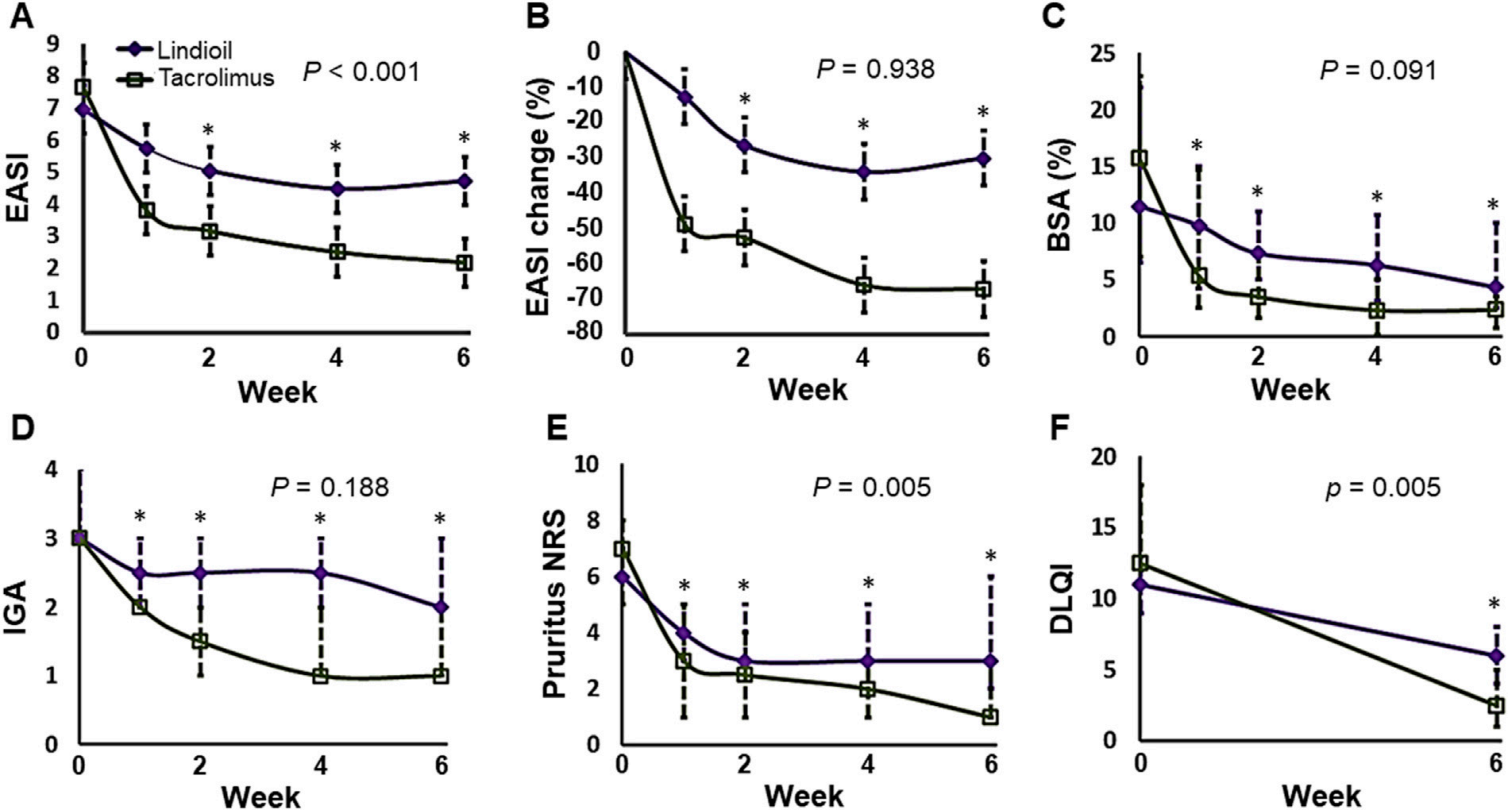
Analyses of the efficacy parameters in the Lindioil and tacrolimus treatment groups at baseline (week 0), week 1, week 2, week 4, and week 6. **(A)** Estimated EASI. **(B)** Estimated EASI percentage change (%). **(C)** BSA percentage change (%). **(D)** IGA. **(E)** Pruritus NRS. **(F)** DLQI. EASI, eczema areas severity index; BSA, body surface area; IGA, investigator’s global assessment; DLQI, dermatology life quality index. Statistical significance: P < 0.05; *P < 0.05 when compared to baseline by linear mixed effect model or Wilcoxon signed-rank test.

The PP population was the primary population for all efficacy analyses. The efficacy parameters were evaluated at baseline, week 1, week 2, week 4, and week 6. Evaluation of the primary efficacy endpoint showed that after 6 weeks of treatment, in the Lindioil group the EASI decreased significantly (a decrease of 1.9, P = 0.017), and it decreased (a decrease of 4.1, P < 0.001) in the tacrolimus ointment group (Figure 2; Table 2). In addition, the EASI percentage change was −40.7% vs. −71.3% for Lindioil ointment vs. tacrolimus ointment (P = 0.017) (Figure 2; Table 2). The linear mixed effect model showed that patients treated with Lindioil ointment had an estimated EASI score of 7.0 (95% CI: 5.5, 8.5) at baseline, which decreased to 4.7 (95% CI: 3.2, 6.2) at week 6, and patients treated with tacrolimus ointment had an estimated EASI of 7.7 (95% CI: 6.1, 9.2) at baseline, which decreased to 2.2 (95% CI: 0.7, 3.7) at week 6. The mean difference in EASI score between treatments was 1.5 (95% CI: 0.9, 2.2) (P < 0.001, Figure 2A). The estimated percentage change in EASI score at week 6 was −30.1% (95% CI: -45.8, −14.5) in the Lindioil group and −67.2% (95% CI: -82.9, −51.6) in the tacrolimus group (Figure 2B). The mean difference in percentage change of EASI score between treatments was 26.3 (95% CI: 16.7, 35.8) over the margin of 19%, and could not reject the non-inferior hypothesis (P = 0.938, Figure 2B).

**TABLE 2:** Treatment outcomes.

	Lindioil (n=14)	Tacrolimus (n=14)	P-value
EASI at baseline	6.6 (3.1, 15.6)	6.7 (1.8, 16.6)	0.626
EASI at week 6	3.4 (0, 12.3)	1.9 (0, 6)	<0.001*
EASI change at week 6	−1.9 (−6.8, 5.7)^#^	−4.1 (−12.5, 0.1)^#^	0.049*
EASI change % at week 6	−40.7 (−100, 87)	−71.3 (−100, 5.7)	0.017*
EASI improvement, n (%)			
EASI-50	5 (35.7)	12 (85.7)	0.039*
EASI-75	1 (7.1)	6 (42.9)	0.063
EASI-90	1 (7.1)	2 (14.3)	1.000
IGA 0 or 1 at week 6	4 (28.6)	9 (64.3)	0.125
IGA change at week 6	−1 (−3, 0)^#^	−2 (−3, 0)^#^	0.188
≥2-point improvement, n (%)	4 (28.6)	9 (64.3)	0.125
BSA change at week 6	−4.4 (−20.8, 5.0)^#^	−9.8 (−29.0, −2.2)^#^	0.091
BSA change % at week 6	−43.6 (−100.0, 53.8)	−86.7 (−100.0, −12.9)	0.002*
Pruritus NRS change at week 6	−2.5 (−6, 6)^#^	−5.5 (−7, −1)^#^	0.005*
≥3-point improvement, n (%)	7 (50.0)	12 (85.7)	0.125
DLQI change at week 6	−5 (−10, 5)^#^	−10 (−15, 0)^#^	0.005*
≥4-point improvement, n (%)	8 (57.1)	12 (85.7)	0.219
SGA at week 6			
SGA 0 or 1, n (%)	6 (42.9)	10 (71.4)	0.289
Drug free day during follow-up			
IGA 0 or 1 at week 6 (n = 3)	44 (23, 49)	28 (10, 56)	0.500

AD, atopic dermatitis; BMI, body mass index; BSA, body surface area involved; EASI, eczema area and severity index; IGA, Investigator’s Global Assessment; NRS, numerical rating scale; DLQI, Dermatology Life Quality Index; SGA, Subject’s Global Assessment. P < 0.001 for the comparison of the difference from baseline by Wilcoxon signed-rank test. *p < 0.05. ^#^ *p* < 0.05 for the comparison of the difference from baseline by Wilcoxon signed-rank test.

For the secondary efficacy endpoints, the results showed that after 6 weeks of treatment the EASI-50, EASI-75, and EASI-90 for Lindioil ointment vs. tacrolimus ointment was 36% vs. 86% (P = 0.039), 7% vs. 43% (P = 0.063), and 7% vs. 14% (P = 1.000), respectively. With respect to IGA, there was no significant difference between the 2 study groups in the median IGA change (P = 0.188), number of patients achieving complete or near complete clearance (IGA = 0 or 1) (P = 0.125), and IGA ≥ 2-point improvement (P = 0.125). There was no significant difference between the median BSA change (P = 0.091) between the groups, but the percentage change was significantly lower in the tacrolimus group (−43.6% vs. −86.7%, P = 0.002). The median difference in decrease of pruritus NRS between the groups was significantly different (−2.5 vs. −5.5 for Lindioil ointment vs. tacrolimus ointment, P = 0.005); however, there was no significant difference in the pruritus NRS ≥ 3-point improvement (P = 0.125). There was statistically significant difference in the median DLQI change (−5 vs. −10 for Lindioil ointment vs. tacrolimus ointment, P = 0.005), but there was no significant difference between in the DLQI ≥ 4-point improvement (P = 0.219) (Figure 2; Table 2).

After treatment, the number of patients achieving SGA = 0 or 1 was not significantly different between the groups [6 (43%) vs. 10 (71%), P = 0.289]. In addition, only 3 patients achieved IGA = 0 or 1 after both study treatments, and the drug free duration at follow-up was not significantly different between the groups (44 vs. 28 days, P = 0.500) (Supplementary Figure S1).

In brief summary, both Lindioil ointment and tacrolimus ointment 0.1% effectively alleviated AD symptoms. Most of outcomes, including EASI change, EASI-50, BSA percentage change, pruritus NRS change and DLQI change, achieved a statistically significant difference after 6 weeks of treatment. While there were no significant differences between the 2 study groups in other secondary efficacy endpoints, including EASI-75, EASI-90, IGA change and SGA change. In addition, tacrolimus ointment 0.1% is slightly superior compared with Lindioil ointment with respect to effectiveness for treating AD, but the drug free duration at follow-up is slightly longer for Lindioil ointment compared with tacrolimus ointment 0.1%.

Efficacy analysis after the first treatment was performed using the safety population (Supplementary Table S1). After 6 weeks of treatment, there was no significant difference in EASI assessments: EASI change percentage was −43% vs. −73% for Lindioil ointment vs. tacrolimus ointment (P = 0.131), and the EASI-50, EASI-75, and EASI-90 for Lindioil ointment vs. tacrolimus ointment was 45% vs. 73% (P = 0.387), 18% vs. 45% (P = 0.361), and 9% vs. 9% (P = 1.000). There was no significant difference between the 2 study groups in the number of patients achieving IGA = 0 or 1 (27% vs. 45%, P = 0.387), but there was significant difference in the number of patients achieving IGA ≥ 2-point improvement (27% vs. 73%, P = 0.033). BSA percent change was −42% in the Lindioil ointment group and −89% in the tacrolimus ointment group (P = 0.101). The change of pruritus NRS was significantly different between the groups (−2 vs. −4, P = 0.039), but the ≥ 3-point improvement in pruritus NRS was not significantly different (5 vs. 9, P = 0.183). The change of DLQI was significantly different between the groups (−2 vs. −11, P = 0.010), but the ≥ 4-point improvement in DLQI was not (5 vs. 8, P = 0.387). There was no difference in SGA = 0 or 1 at week 6 (P = 0.361). The drug free duration at follow-up for patients achieving IGA = 0 or 1 was not statistically different between the groups (56 vs. 15 days, P = 0.052). In brief, tacrolimus 0.1% is slightly superior compared with Lindioil ointment in effectiveness for treating AD, but the drug free duration at follow-up is slightly longer for Lindioil ointment compared with tacrolimus ointment 0.1%.

**FIGURE 3:**
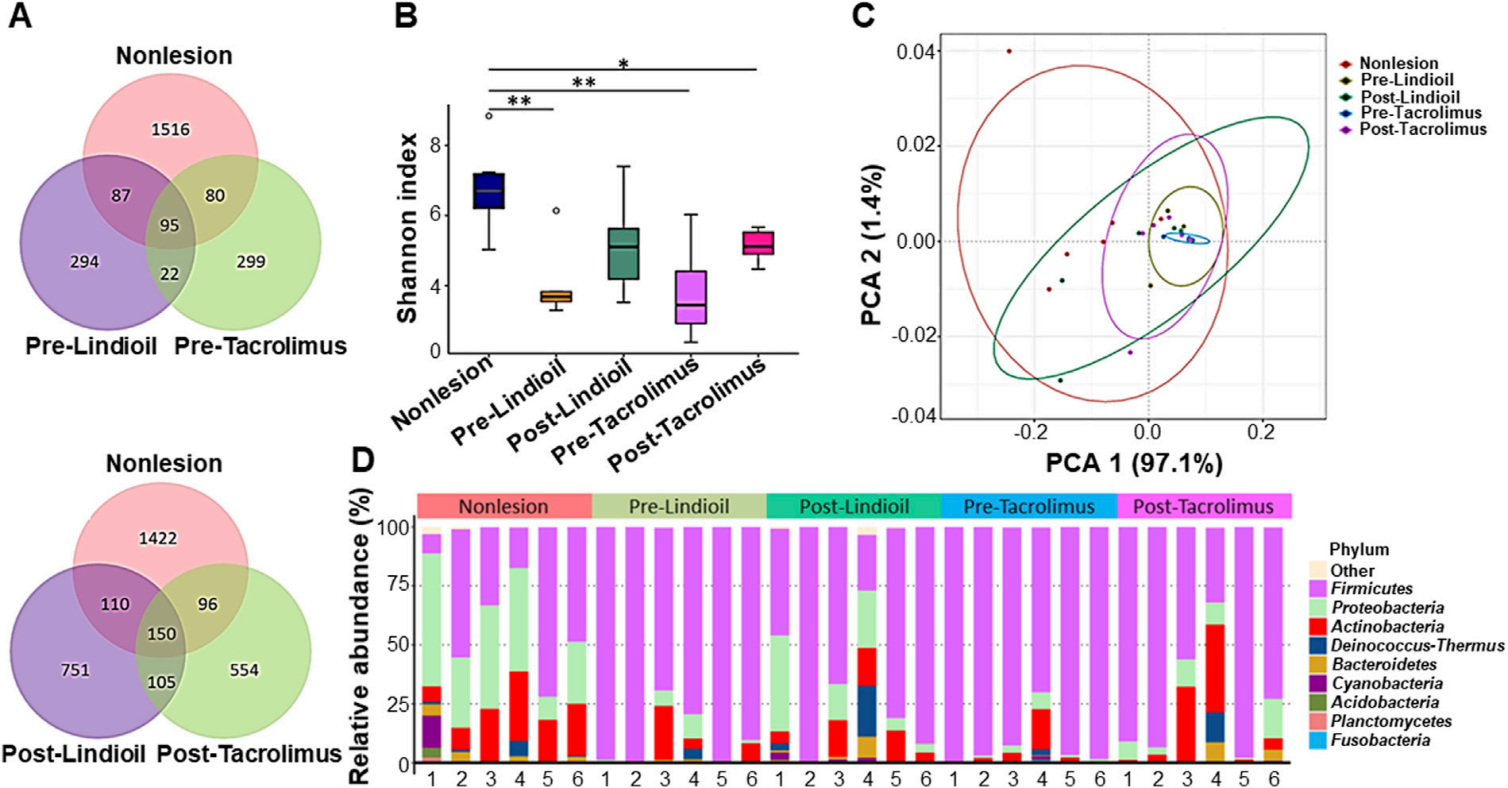
Diversity analyses of the skin microbiota pre- and post- Lindioil or tacrolimus treatment. **(A)** Venn diagram. **(B)** Box-and-whisker plot of Shannon diversity index. **(C)** Weighted UniFrac principal coordinates analysis (PCoA) plot. **(D)** Relative abundance bar chart illustrating the top 10 phyla of skin microbiota species.

For skin microbiota analysis, 16S rRNA sequence analysis was performed to identify the strains of microbiota isolated from the most severe lesion area before treatment, the corresponding area after treatment, and non-lesion areas located at least 10 cm away from the lesion areas. The Venn diagrams based on the error-corrected ASV showed an increase in species richness after treatment with Lindioil ointment or tacrolimus ointment, along with an elevated intersection of species with the non-lesion area (Figure 3A). The Shannon diversity index showed that the strain diversity in the non-lesion areas was significantly different from the species diversity in pre-treatment lesion areas treated with Lindioil or tacrolimus ointment (P < 0.01), and in the tacrolimus post-treatment lesion area (P < 0.05) (Figure 3B). A weighted UniFrac principal coordinates analysis (PCoA) based on species abundance data was plotted, and analysis of similarities (ANOSIM) revealed significant dissimilarities between the non-lesion skin and Lindioil ointment pre-treatment areas (R = 0.59, P = 0.005), tacrolimus pre-treatment areas (R = 0.67, P = 0.001), and tacrolimus post-treatment areas (R = 0.41, P = 0.011). However, there was no significant difference between non-lesion and Lindioil post-treatment areas (R = 0.08, P = 0.190), Lindioil pre- and post-treatment areas (R = 0.08, P = 0.147), tacrolimus pre- and post-treatment areas (R = 0.17, P = 0.095), and Lindioil and tacrolimus pre-treatment areas (R = −0.06, P = 0.570), and Lindioil and tacrolimus post-treatment areas (R = −0.02, P = 0.517) (Figure 3C).

**FIGURE 4:**
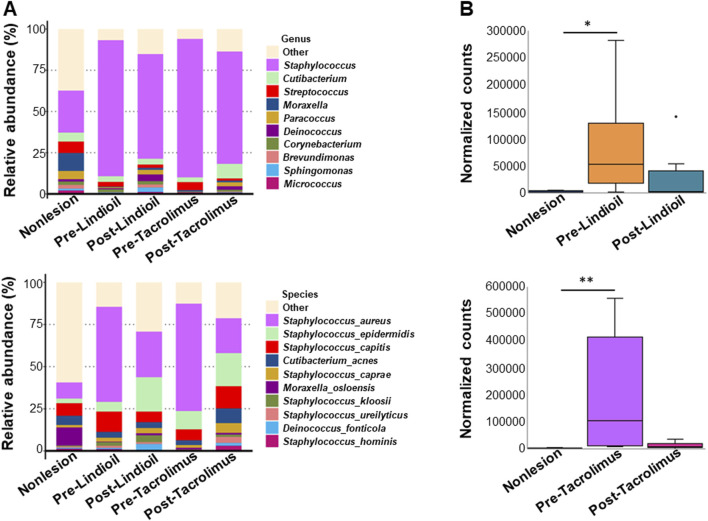
Relative abundance of the genus and species of skin microbiota pre- and post-treatment. **(A)** Bar chart showing the relative abundance (%) of genus (upper) and the species (lower). **(B)** Box-and-whisker plot showing the normalized counts of genus (upper) and the species (lower).

The species relative abundance bar chart illustrating the top 10 phyla indicated a shift from *Firmicutes* dominance in AD lesions pre-treatment to an increased abundance of *Proteobacteria* post-treatment, resembling that of non-lesion skin (Figure 3D). Genus and species-level relative abundance charts showed that compared to non-lesion skin, *S. aureus* was predominant in AD lesions pre-treatment. The proportion of *S. aureus* decreased post-treatment, with no significant difference between the 2 treatments. Pre-treatment *S. aureus* levels were significantly different than in non-lesion skin for both treatments (P = 0.015, P = 0.003), and post-treatment *S. aureus* levels were decreased after both treatments, and there was no significant difference from non-lesion skin or between the 2 treatments (Figures 4A,B).

Most patients (71%) considered tacrolimus ointment 0.1% to be more effective, but had more side-effects; however, no significant difference in the sequence of the 2 treatments was noted (Supplementary Table S2). Results of the chi-square goodness-of-fit analysis showed that there was no significant difference of efficacy or side-effects between the 2 treatments (P = 0.109). Evaluation of the most bothersome side effects revealed that pruritus as the primary concern for patients using Lindioil ointment (2/4, 50%), while those using tacrolimus ointment indicated that a burning sensation was the most bothersome side effect (4/10, 40%).

### 3.3 Safety results

A comprehensive overview of adverse events is presented in Table 3. Over the 29-week trial period, a total of 19 patients (86%) experienced 56 adverse events. Among these, 12 (54%) encountered 24 local adverse reactions. Severity assessment showed that the majority of adverse events were classified as mild, and 4 (18%) were of moderate intensity. Notably, no severe adverse events occurred, and no adverse events necessitated the discontinuation of treatment.

**TABLE 3:** Adverse events (AEs).

	Lindioil (n = 22)	Tacrolimus (n = 18)
Patients with AEs, n (%)	9 (40.9)	18 (100.0)
Patients with SAEs, n (%)	0 (0.0)	0 (0.0)
Patients with ASRs, n (%)	2 (9.1)	11 (61.1)
Total AEs	18	38
Total SAEs	0	0
Total ASRs	4	20
AEs by severity, n (%)		
Mild	14, 8 (36.4)	37, 18 (100.0)
Moderate	4, 3 (13.6)	1, 1 (5.6)
Severe	0 (0.0)	0 (0.0)
AEs by causality, n (%)		
Unrelated/Unlikely	12, 8 (36.4)	16, 11 (61.1)
Possible/Probable	2, 2 (9.1)	2, 2 (11.1)
Highly probable	4, 2 (9.1)	20, 11 (61.1)

SAE, serious adverse event; ASR, application site reaction.

Compliance with clinical interventions was assessed by calculating the actual number of days patients used the ointments divided by the prescribed number of days, multiplied by 100 (Supplementary Table S3). Irrespective of the treatment period, there were no significant differences in compliance between the 2 treatment groups for all study populations. However, a significant difference in compliance between the 2 treatments was observed in the second treatment phase of the PP population (P = 0.021).

## 4 Discussion

This evaluator-blinded, randomized study was designed to compare the efficacy and safety of Lindioil ointment and tacrolimus ointment 0.1% for the treatment of mild-to-severe AD. The results showed that both Lindioil ointment and tacrolimus ointment 0.1% effectively alleviated AD symptoms. In addition, both treatments showed favorable safety profiles and patient tolerability, with no severe adverse events observed. Notably, the key findings of the study are that Lindioil ointment significantly improves AD severity (EASI, IGA, and pruritus NRS) and DLQI. Also important, the abundance of the AD severity-associated skin microbe *S. aureus* at AD lesions was reduced by local application of Lindioil ointment, with a similar abundance between post-treatment AD lesions and non-lesion skin.

Significant improvements have been observed in the EASI, IGA, pruritus NRS, and DLQI in patients with moderate-to-severe AD after treatment with tacrolimus ointment for 4 weeks (Won et al., 2004). A similar observation was noted in our patients with mild-to-severe AD after tacrolimus ointment treatment for 6 weeks. Lin et al. (2020) reported a significant decrease in EASI score in patients with AD treated with Lindioil ointment, a result also seen in our study. Notably, the present study expanded the findings of the prior study and demonstrated significant improvements in other indexes of AD severity (IGA, BSA, and pruritus NRS) and DLQI in patients treated with Lindioil ointment for 6 weeks. Although tacrolimus ointment showed a more favorable overall efficacy compared with Lindioil ointment, only 10% patients using Lindioil ointment experienced localized adverse reactions, while approximately 60% of those using tacrolimus ointment reported such reactions.

The skin *Firmicutes* phylum and *S. aureus* species are dominant in moderate AD, while the skin *Proteobacteria* phylum is dominant in mild AD (Suwarsa et al., 2021). Similarly, the dominance of the *Firmicutes* phylum and *S. aureus* species in skin lesions was observed in our population with mild-to-severe AD. In particular, we demonstrated a skin microbiota shift from the dominance of the *Firmicutes* phylum in AD lesions pre-treatment to an increased abundance of *Proteobacteria* phylum in AD lesions post-treatment with Lindioil ointment or tacrolimus ointment; abundances similar to those of non-lesion skin. In addition, the proportion of *S. aureus* species in AD lesions significantly decreased after Lindioil ointment or tacrolimus ointment treatment, showing no significant differences from non-lesion skin or between the 2 treatments. To the best of our knowledge, the present study is the first to report that local application of Lindioil ointment reduces the abundance of *S. aureus* at AD lesions, a bacterium known for its significant association with AD severity (Clausen et al., 2017; Khadka et al., 2021). These findings suggest that Lindioil ointment may act as a prebiotic to alleviate AD by altering skin microbiota.

AD is believed to be primarily due to an impaired epidermal barrier function and immune function disorder in the skin. Recent studies have revealed that genetic mutations in filaggrin, an epidermal protein that plays a crucial role in maintaining skin structure and function, result in defects in the barrier function of the skin stratum corneum, which leads to increased transepidermal water loss (Drislane and Irvine, 2020). This, in turn, facilitates the entry of antigens into the epidermis, triggering an immune response and causing infiltration of inflammatory cells into acute AD lesions. A large number of T cells, including Th_2_ and Th_22_ cells, as well as smaller proportions of Th_1_ and Th_17_ T cells, are activated leading to the release of proinflammatory and pruritic substances. This process can damage epidermal differentiation and integrity, and keratinocytes. Upon progression into the chronic phase, abnormal proliferation and differentiation of the epidermis occurs, and sustained activation of immune cells further compromise the skin barrier function. Consequently, symptoms of skin inflammation persistently recur over a long period of time. Furthermore, studies have found that filaggrin mutations contribute to increased colonization of skin *S. aureus*. Patients with both filaggrin mutations and colonization of *S. aureus* exhibit higher severity scores in the Scoring Atopic Dermatitis (SCORAD) assessment, indicating a more severe manifestation of AD (Clausen et al., 2017). While the main components of Lindioil ointment, Qingdai and indirubin, demonstrate antimicrobial properties by inhibiting the growth of Gram-positive bacteria, including *S. aureus*, as well as fungi such as *A. fumigates* and *C. albicans* (Ponnusamy et al., 2010; Chiang et al., 2013; Gaitanis et al., 2019). For this aspect, we further demonstrated that pre-treatment *S. aureus* levels were significant differences when compared to non-lesion skin for both Lindioil ointment and tacrolimus ointment. Following treatment, the post-treatment *S. aureus* levels were reduced for both treatments, with no significant differences observed in comparison to non-lesion skin. However, tacrolimus ointment had a greater reduction in post-treatment *S. aureus* levels than did Lindioil ointment, although it was not statistically different. Consequently, we speculated that the suppression of *S. aureus* colonization at AD lesions may be one of the possible mechanisms by which Lindioil and tacrolimus ointments ameliorates AD. Tacrolimus ointment may be more therapeutic efficacy for improving AD symptoms due to a greater reduction of skin *S. aureus*.

Pharmacological studies have shown that both Qingdai and indirubin can regulate excessive proliferation and abnormal differentiation of keratinocytes (Lin et al., 2009a; Hsieh et al., 2012), and can enhance expression of claudin-1, thus improving epidermal barrier function (Lin et al., 2013). In addition, they exhibit anti-oxidative stress, anti-inflammatory, and immunomodulatory effects, and thus reduce the generation or enhance the elimination of reactive oxygen species (ROS) (Lin et al., 2009b; Ahmad et al., 2010; Lin et al., 2012b; Zhao et al., 2017). This is believed to be achieved by decreasing levels of pro-inflammatory cytokines such as TNF-α, IL-6, IFN-γ, and IL-17 (Kim et al., 2013; Gao et al., 2016; Cheng et al., 2017; Xie et al., 2018; Yang et al., 2021), or increasing levels of anti-inflammatory cytokines like IL-10 (Zhang et al., 2015; Kawai et al., 2017). Indirubin can decrease serum immunoglobulin E (IgE) concentrations, and enhance the production of Foxp3 regulatory T cells (Gao et al., 2016; Zhang et al., 2015). The pathogenesis of AD includes skin barrier dysfunction and alterations of immunity (Sroka-Tomaszewska and Trzeciak, 2021); therefore, we also speculate that another possible mechanism by which Lindioil is effective for treating AD is restoring skin barrier dysfunction and immunity.

There are several limitations to this study that should be considered. First, the number of patients in the study was small due to the COVID-19 pandemic. This may impact on the interpretation of results, hence there is the need for larger scale research in the future to validate the conclusions. Second, considering the susceptibility of evaluation outcomes to extreme values, and the influence of environmental factors such as air pollution, volatile organic compounds, and allergens on the symptoms of AD, caution is warranted in interpreting the findings (Weidinger et al., 2018). Third, over 50% of patients in the present study had sensitivities to substances like food and dust mites, implying that inadvertent exposure to these allergic triggers before follow-up visits could exacerbate symptoms, potentially influencing treatment outcomes.

## 5 Conclusion

Lindioil ointment is effective and safe for treating mild-to-severe AD, making it a viable and safe alternative for patients with concerns about the safety or tolerability of tacrolimus. Notably, Lindioil ointment alters the skin microbial composition, suggesting the possible use of Lindioil ointment as a prebiotic to alleviate AD. Further studies with larger sample sizes and extended observation periods are warranted to confirm these preliminary findings, and enhance the robustness of the conclusions.

## Data Availability

The data presented in the study are deposited in the Figshare repository, available at https://figshare.com/s/40c409d545373c3acd42.
