# Supplementary material for: Efficacy and safety of indigo naturalis oil extract (Lindioil ointment) for the treatment of atopic dermatitis: a randomized, crossover, evaluator-blinded, controlled trial
Source: Front Pharmacol. 2025 Jul 8;16:1546589. doi: 10.3389/fphar.2025.1546589 (PMC12279832; doi:10.3389/fphar.2025.1546589)

**Efficacy and safety of indigo naturalis oil extract (Lindioil ointment) for the treatment of atopic dermatitis: a randomized, crossover, evaluator-blinded, controlled trial**

**Running title:** Lindioil ointment for AD treatment

**Supplementary Table 1. Efficacy of Lindioil and tacrolimus after first treatment period**

|  | **Lindioil (n=11)** | **Tacrolimus (n=11)** | **P-value** |
| --- | --- | --- | --- |
| **EASI** |  |  |  |
| Change at week 6, median (range) | -2.8 (-6.5, 0.5)**^#^** | -5.5 (-11.8, -1.8)**^#^** | 0.022* |
| % Change at week 6, median (range) | -43.3 (-100.0, 6.8) | -72.9 (-91.2, -39.4) | 0.131 |
| EASI-50 at week 6, n (%) | 5 (45.5) | 8 (72.7) | 0.387 |
| EASI-75 at week 6, n (%) | 2 (18.2) | 5 (45.5) | 0.361 |
| EASI-90 at week 6, n (%) | 1 (9.1) | 1 (9.1) | 1.000 |
| **IGA** |  |  |  |
| IGA 0 or 1 at week 6, n (%) | 3 (27.3) | 6 (54.5) | 0.387 |
| Change at week 6, median (range) | -1 (-3, 0)**^#^** | -2 (-2, 0)**^#^** | 0.208 |
| ≥ 2-point improvement at week 6, n (%) | 3 (27.3) | 8 (72.7) | 0.033* |
| **BSA** |  |  |  |
| Change at week 6, median (range) | -5 (-22.8, 3.5)**^#^** | -13.3 (-29.0, -4.3)**^#^** | 0.023* |
| % Change at week 6, median (range) | -42.5 (-100.0, -53.8) | -88.6 (-98.8, -12.9) | 0.101 |
| **Pruritus NRS** |  |  |  |
| Change at week 6, median (range) | -2 (-6, 6) | -4 (-7, -1)**^#^** | 0.039* |
| ≥ 3-point improvement at week 6, n (%) | 5 (45.5) | 9 (81.8) | 0.183 |
| **DLQI** |  |  |  |
| Change at week 6, median (range) | -2 (-10, 7) | -11 (-15, -2)**^#^** | 0.010* |
| ≥ 4-point improvement at week 6, n (%) | 5 (45.5) | 8 (72.7) | 0.387 |
| **SGA**, n (%) |  |  |  |
| SGA 0 or 1 at week 6 | 6 (54.5) | 9 (81.8) | 0.361 |
| **Drug free day**, median (range) |  |  |  |
| IGA 0 or 1 (n=3, n=6) | 56 (44, 56) | 15 (10, 49) | 0.052 |

BSA, body surface area involved; EASI, Eczema Area and Severity Index; IGA, Investigator’s Global Assessment; NRS, Numerical Rating Scale; DLQI, Dermatology Life Quality Index. SGA, Subject’s Global Assessment. P<0.001 for the comparison of the difference from baseline by Wilcoxon signed-rank test. *P<0.05; ^#^P<0.05 for the comparison of the difference from baseline by Wilcoxon signed-rank test.

**Supplementary Table 2. Patient treatment preferences**

|  | **Lindioil first (n=7)** | **Tacrolimus first**  **(n=7)** | **P-value^1^** | **P-value^2^** |
| --- | --- | --- | --- | --- |
| **Better efficacy** |  |  |  |  |
| Lindioil | 2 (28.6) | 2 (28.6) | 1.000 | 0.109 |
| Tacrolimus | 5 (71.4) | 5 (71.4) |  |  |
| **More side effects** |  |  |  |  |
| Lindioil | 2 (28.6) | 2 (28.6) | 1.000 | 0.109 |
| Tacrolimus | 5 (71.4) | 5 (71.4) |  |  |

^1^P-values are determined by Fisher’s exact test.

^2^P-values are determined by chi-square goodness-of-fit test for the predicted probability of Lindioil and Tacrolimus as 0.5 vs 0.5 in all participants.

**Supplementary Table 3. Treatment compliance**

| **Treatment period** | **Lindioil** | **Tacrolimus** | **P-value** |
| --- | --- | --- | --- |
| **Safety population** | n=22/11/11 | n=18/11/7 |  |
| All periods | 97.7 (66.7, 100.0) | 100.0 (90.7, 100.0) | 0.199 |
| Period 1 | 98.0 (86.1, 100.0) | 97.7 (91.1, 100.0) | 0.863 |
| Period 2 | 97.7 (66.7, 100.0) | 100.0 (90.7, 100.0) | 0.087 |
| **ITT population** | n=18/7/11 | n=18/11/7 |  |
| All periods | 97.7 (66.7, 100.0) | 100.0 (90.7, 100.0) | 0.195 |
| Period 1 | 98.0 (86.8, 100.0) | 97.7 (91.1, 100.0) | 0.962 |
| Period 2 | 97.7 (66.7, 100.0) | 100.0 (90.7, 100.0) | 0.087 |
| **PP population** | n=14/7/7 | n=14/7/7 |  |
| All periods | 96.5 (86.8, 100.0) | 99 (90.7, 100.0) | 0.150 |
| Period 1 | 98.0 (86.8, 100.0) | 97.7 (93, 100.0) | 0.793 |
| Period 2 | 95.3 (90.7, 97.7) | 100.0 (90.7, 100.0) | 0.021* |

**Supplementary Figure 1.** The number of patients achieving SGA = 0 or 1, and the drug free duration (days) at follow-up. SGA, subject global assessment.


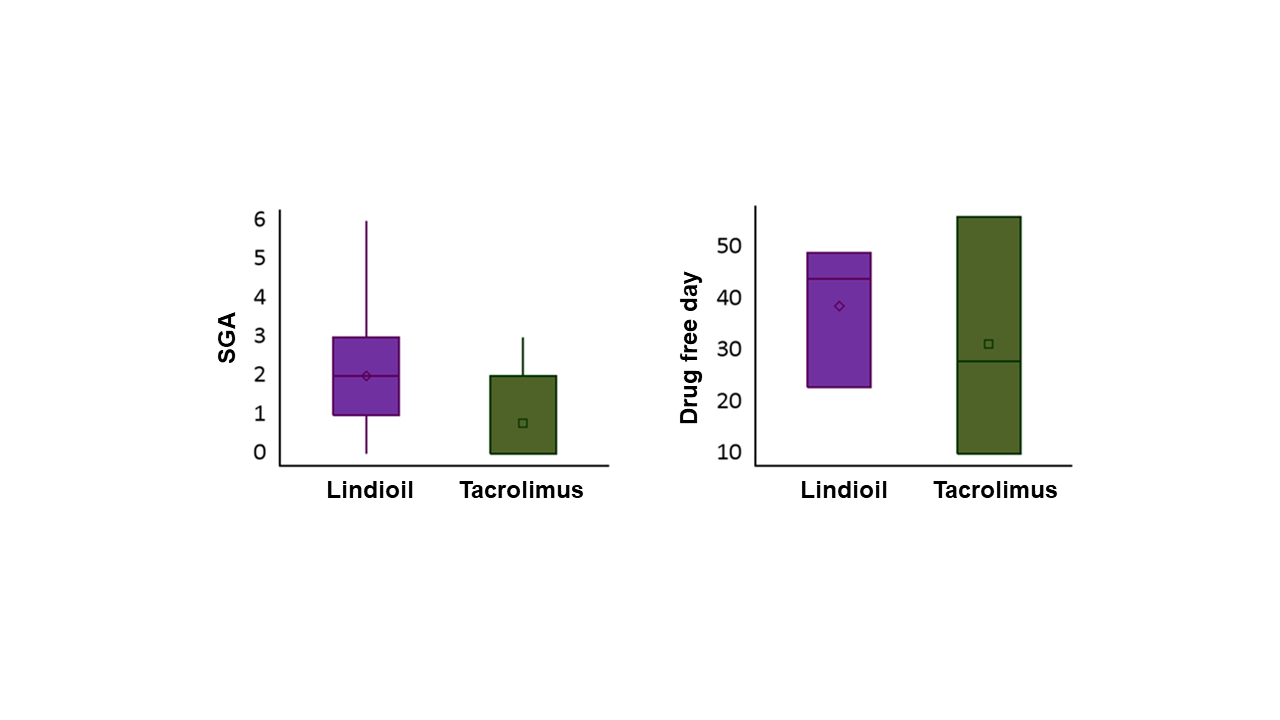

Supplement: Supplementary file 1 [file Supplementaryfile1.docx]
